# Beyond a one-size-fits-all approach: the asymmetrical roles of personality and social support in promoting outdoor physical activity

**DOI:** 10.3389/fpubh.2026.1820567

**Published:** 2026-07-15

**Authors:** Kunpeng Li, Marcin Białas, Mingzhu Wu

**Affiliations:** 1 Faculty of Physical Culture, Gdansk University of Physical Education and Sport, Gdansk, Poland; 2 Gdansk University of Physical Education and Sport, Gdansk, Poland

**Keywords:** basic psychological needs, exercise enjoyment, outdoor physical activity, personality traits, social support

## Abstract

**Purpose:**

Recognizing outdoor exercise enjoyment as a vital protective factor for public mental health, this study constructed a moderated mediation model to examine the associations between personality traits (specifically extraversion and neuroticism) and outdoor exercise enjoyment. The investigation focused on the dual mediating roles of basic psychological need satisfaction and frustration. Furthermore, it explored the boundary conditions of social support to determine how environmental factors interact with individual dispositions.

**Methods:**

A cross-sectional survey was conducted with 527 outdoor exercise participants in first-tier cities in China. Moderated mediation models were examined for both extraversion and neuroticism to enjoyment through basic psychological need satisfaction and frustration, as well as to determine the moderation of social support on this relationship.

**Results:**

Evidence to support an asymmetrical dual mediation mechanism was confirmed; extraversion largely supported enjoyment through psychological need satisfaction, while neuroticism detracted from enjoyment primarily through psychological need frustration. Social support demonstrated complicated moderation patterns; social support strengthened the positive social association of extraversion with psychological need satisfaction (a gain dynamic), yet, in the neuroticism group we observed a paradox. Specifically, while high social support did buffer against the detriment of neuroticism on psychological need satisfaction, it increased the positive association of neuroticism and psychological need frustration.

**Conclusions:**

Social support is not an absolute remedy, but is conditional on personality traits and can be a stressor under certain conditions. By delineating these boundaries within the Person-Environment Fit framework, this study highlights the necessity for differentiated intervention strategies. We propose different intervention approaches: strengthening social connections for extraverted individuals, and providing non-judgmental non-invasive support for neurotic individuals to break the frustration link, thereby informing targeted public mental health services and mitigating psychological distress.

## 1 Introduction

Finding pleasure in movement forms the fundamental basis of exercise adherence and long-term health outcomes (1, 2). When individuals have pleasurable and positive experiences while exercising, they are more likely to report higher levels of intrinsic motivation, which translates into consistent physical activity routines and subsequent psychophysical wellbeing (3, 4). In contrast, a lack of enjoyment is associated with detrimental attitudes, lower involvement, and activity discontinuation, precluding individuals from realizing the health benefits of physical activity (5). Consequently, understanding the psychological ingredients of exercise enjoyment is valuable on a public health level. As outdoor physical activities rapidly increase, exploring the specific psychological mechanisms that promote exercise enjoyment in nature—an environment known to offer unique psychological benefits beyond indoor settings (6)—is increasingly necessary.

To understand the mechanisms underpinning exercise enjoyment, Self-Determination Theory (SDT) provides a robust theoretical framework. SDT asserts that the basic psychological needs for autonomy, competence, and relatedness are the primary correlates of intrinsic motivation and positive affect (7, 8). Recently, researchers have advanced this framework by conceptualizing basic psychological need satisfaction (BPNS) and need frustration (BPNF) as distinct constructs rather than opposite ends of a single continuum (9, 10). Whereas basic needs satisfaction represents the bright side of human experience—promoting vitality and enjoyment in physical activity (11, 12), psychological need frustration represents the dark side. Empirical evidence suggests that needs frustration is actively linked to feelings of isolation, incompetence, and maladaptive outcomes, demonstrating a stronger negative association than mere low needs satisfaction (9). Although this dual-process perspective is critical for explaining why some individuals thrive in outdoor activities while others feel distressed, it has been largely overlooked in the existing exercise enjoyment literature.

Importantly, these basic psychological needs are not experienced uniformly; individual differences, particularly personality traits, play a critical role in shaping how individuals appraise and emotionally respond to exercise contexts (1, 13). Extant literature suggests that among the Big Five, extraversion and neuroticism uniquely demonstrate the most robust and symmetrical logical alignment with affective responses within the SDT framework (14, 15). Driven by the behavioral approach system and reward sensitivity, extraversion is intrinsically linked to positive engagement and the active seeking of social connection and challenges, thus facilitating psychological need satisfaction (16, 17). Conversely, neuroticism is driven by the behavioral avoidance system and threat sensitivity. Individuals higher in neuroticism are more susceptible to perceiving exercise environments as uncontrollable or threatening, which actively triggers psychological need frustration (18, 19). Despite these theoretical frameworks linking extraversion and neuroticism to exercise enjoyment via the asymmetrical processes of satisfaction and frustration, limited research has examined these dual links simultaneously.

Furthermore, the interplay between personality and psychological needs does not occur in a vacuum; environmental contexts, particularly social support, establish critical boundary conditions (20). Generally, social support from family and friends serves as a salient resource that facilitates needs satisfaction and acts as a protective buffer against needs frustration (21, 22). Relying on the traditional stress-buffering hypothesis, social support is often assumed to be universally beneficial, mitigating distress for vulnerable individuals while enhancing the wellbeing of socially oriented individuals (23). However, the dynamic interaction between personality traits and social support in outdoor exercise contexts remains ambiguous. It is still unclear whether social support unequivocally exacerbates the positive associations of extraversion while buffering the negative associations of neuroticism. Emerging perspectives suggest that support must be carefully evaluated for its qualitative features (e.g., autonomy-supportive vs. controlling); inappropriate or high-pressure support might inadvertently elicit social evaluation anxiety or introjected regulation, potentially turning a perceived resource into a stressor for sensitive individuals.

**Figure 1:**
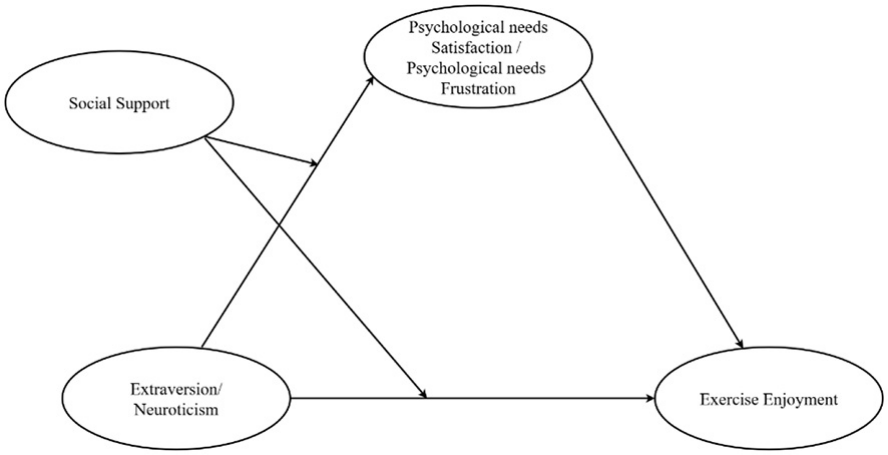
Conceptual model.

To bridge these overarching research gaps, the present study aims to construct an integrative moderated mediation model within the context of outdoor physical activity. The conceptual model is presented in Figure 1. Specifically, this research seeks to: (1) examine the direct associations of extraversion and neuroticism with outdoor exercise enjoyment; (2) explore the distinct, asymmetrical mediating roles of psychological need satisfaction and psychological need frustration; and (3) investigate the moderating role of social support on the relationships between personality traits and psychological needs. By dissecting these dual processes, this study aims to provide empirical insights to inform the development of more tailored, personality-aware guidance strategies in outdoor exercise settings. Crucially, this study seeks to challenge the traditional one-size-fits-all assumption of social support, exploring whether it acts as a double-edged sword that might manifest as an unexpected stressor for neurotic individuals in certain cultural contexts.

## 2 Materials and methods

### 2.1 Study procedures and participants

This research utilized a cross-sectional survey design. To address significant cultural and economic differences at the regional level, Beijing, Shanghai, and Guangzhou (representing Northern China, Eastern China, and Southern China as core cities) were chosen as the empirical settings. The time frame for data collection was between June and August 2025, using the scale and reach of the online platform Wenjuanxing due to its large user pool. Eligibility for participation was restricted to permanent residents who had lived in those cities for at least six consecutive months, ensuring they could provide answers based on stable cognitive experiences regarding the local sports context. To support the reliability and validity of the data, a multi-level quality control procedure was put in place: (1) technical screening to prevent duplicate responses based on IP address uniqueness; (2) duration screening to automatically eliminate outlier samples with response durations far below normal; and (3) logical screening, using logical questions and Attention Check Items, for detecting and removing invalid sample data.

The sample selected for the study consisted of people with outdoor exercise experience. The selection of outdoor exercise activities was supported by recognized industry data (China Outdoor Sports Industry Development Report (2023–2024) from Auri and the Xiaohongshu 2023 Outdoor Lifestyle Trend Report) to include mainstream outdoor exercise activities such as hiking, cycling, camping, and water sports. This allows for a high level of ecological validity and connection to current trends in outdoor sports participants' identities. The surveys were administered in two separate phases. The pilot test received 157 valid questionnaires returned. For our theoretically targeted constructs, the pilot test reported Cronbach's α coefficients between 0.708 and 0.915, indicating good reliability. In the formal test, 600 questionnaires were distributed. After cleaning, the final sample consisted of 527 responses.

Regarding geography, the participants were concentrated primarily in Beijing, Shanghai, and Guangzhou and the responses were geographically representative. Slightly more than half the sample population was female (52.6%), and significant proportions of participants were between the ages of 18–40 (86%), followed by 86.3% holding a college degree or higher. Most participants received monthly incomes between 5,000 and 20,000 RMB (77.8%). Preferred outdoor activities included hiking (76.7%), cycling (63.8%), and wilderness camping (66.6%). This study draws upon samples concentrated in first-tier cities in China where participants are educated and the demographic profile of the sample population is inconsistent with the mainstream profile of current participants of outdoor sports.

### 2.2 Instruments and measures

This study used four validated scales to measure personality traits, psychological needs, enjoyment with exercise, and social support. All scales utilized a translation—back-translation—expert review approach standardizing linguistic and cultural adaptation (24).

The Big Five Inventory (BFI-10) shortened version (25) was used to extract targeted personality traits (26). Although this inventory was administered in its entirety to maintain the psychometric validity of the original scale format, based on our *a priori* theoretical framework rooted in Self-Determination Theory (SDT), the current study specifically targeted only two core dimensions: extraversion and neuroticism. As established in the literature, among the Big Five traits, extraversion (driven by the behavioral approach system and reward sensitivity) and neuroticism (driven by the behavioral avoidance system and threat sensitivity) uniquely possess the most robust and symmetrical logical alignment with affective responses (e.g., enjoyment) via the dual pathways of basic psychological needs (14, 15). Therefore, only the four items measuring extraversion and neuroticism from the BFI-10 were utilized for hypothesis testing. Given that Cronbach's α is highly sensitive to the number of items and often underestimates the internal consistency of two-item subscales, we followed standard psychometric recommendations and employed the Spearman-Brown coefficient (ρ_*sb*_) as a more appropriate reliability indicator for these dimensions (27). The empirical data corroborated the reliability of our targeted constructs, yielding excellent internal consistency for both extraversion (ρ_*sb*_ = 0.864) and neuroticism (ρ_*sb*_= 0.911). The choice to focus exclusively on these two core dimensions not only aligns with the fundamental hypotheses of our prediction model but also strictly adheres to their established primacy in the exercise psychology field.

The Basic Psychological Need Satisfaction and Frustration Scale was employed in the study as a quantitative means of measuring the psychological health level of participants (2). The choice for this measurement was theoretically based on SDT. According to SDT, measuring the condition of basic psychological needs is a primary indicator of optimal psychological functioning and individual wellbeing (28). In line with this, the scale has a unique dual-assessment framework, simultaneously measuring the positive experience of psychological need satisfaction, as well as the negative experience of psychological need frustration. This design separably measures the satisfaction and frustration of psychological needs, which allows for the mapping of psychological health in a more complete and multi-dimensional way, compared to a traditional scale measuring one dimension of psychological health. More specifically, the scale measures six sub-dimensions that accurately reflect the experiences of autonomy, relatedness, and competence that occur during outdoor leisure. Descriptive statistics demonstrated the scale had excellent reliability in the study. The level of internal consistency (Cronbach's α) of psychological need satisfaction was 0.887, while psychological need frustration was 0.931.

The Physical Activity Enjoyment Scale –Short Form (PACES-S) is based on the Portuguese version (29, 30) and has been validated into a 4-item scale. For this study, the assessment of enjoyment of exercise was developed using a 7-point bipolar response format. The enjoyment outcome yielded a Cronbach's α value of 0.835, which demonstrates a good level of reliability.

The Perceived Social Support Scale (PSSS-A6) was incorporated to assess an individual's subjective perceptions of social support (31). The primary moderating variables were family support (2 questions) and friend support. This scale uses a seven-point Likert to indicate stronger perceptions of social support within the individual. The reported Cronbach's α values for this sample were 0.808 respectively, consistent with the short 6-item version.

### 2.3 Statistical analysis

For this study, a multi-stage statistical analytic plan was used. The analysis began with the implementation of descriptive statistics and Confirmatory Factor Analysis (CFA), individually, in IBM SPSS 27.0 and AMOS 29.0. These steps were made to establish measurement model fit (32) in combination with reliability and validity estimates. Next, Spearman correlation analysis was used to measure bivariate association among all variables. Hypothesis tests were completed by utilizing the PROCESS macro (v4.2) process developed by Hayes (33). Once the latent variables were converted to mean indicators, hypothesis tests were carried out with Model 4 and Model 8 to disentangle mediation and moderated mediation effects respectively (34). All estimates were based on 5,000 bias corrected bootstrap resampling procedures to guard against bias in our statistical inferences. Estimates were regarded as statistically significant if the 95% confidence intervals (CI) did not include zero (35). We also conducted simple slope analyses to examine the relative strength of the interaction effects, and path differences at the mean and ±1 SD levels.

## 3 Results

### 3.1 Measurement model analysis

To assess discriminant validity among core variable constructs of the study, a series of Confirmatory Factor Analyses (CFA) were performed. We compared the baseline six-factor model (i.e. extraversion, neuroticism, psychological need satisfaction, psychological need frustration, exercise enjoyment, and social support) to five alternative models. Alternative models were created based on a stepwise combination strategy of the specific dimensions based on their very high correlations. The data fit was superior for the six-factor model as presented in Table 1. All fit indices reflect excellent standards recommended for statistical analyses: χ2/*df* = 1.335, GFI = 0.926, AGFI = 0.915, RMSEA = 0.025, SRMR = 0.022, NFI = 0.962, CFI = 0.990 (see Appendix). Again, we note that both RMSEA and SRMR were less than the 0.05 threshold, while CFI and NFI were above 0.950. These estimates demonstrate a strong fit of the theoretical model data (36). In contrast, all alternative models demonstrated poor fit indices. Specifically, the overall fit became worse when extraversion and neuroticism were treated as a single factor (5-factor model). Although some indices were still marginally acceptable (χ2/*df* was overall much larger; RMSEA was 0.056), the overall fit severely declined. A similar worsening occurred for all the alternative fit estimates that were forced combinations. Finally, it's noteworthy that the model had the poorest fit when all variables were loaded into a single factor (1-factor model: χ2/*df* = 24.549, CFI = 0.408, RMSEA = 0.194). Not only does this confirm that our 6-variable constructs have good discriminant validity, but it also suggests that Common Method Bias was not an issue for the current study. Thus, we have established the measurement model as possessing sound construct validity and is suitable for hypothesis testing.

**Table 1:** Measurement models.

		χ2/*df*	GFI	AGFI	RMSEA	SRMR	NFI	CFI
6factor	EX.NE.BPNS.BPNF.EE.SS	1.335	0.926	0.915	0.025	0.0221	0.967	0.990
5factor	EX+NE.BPNS.BPNF.EE.SS	2.977	0.882	0.864	0.056	0.0874	0.928	0.951
4factor	EX+NE.BPNS+BPNF.EE.SS	17.859	0.266	0.169	0.165	0.2273	0.567	0.581
3factor	EX+NE.BPNS+BPNF.EE+SS	20.710	0.251	0.156	0.178	0.2387	0.496	0.507
2factor	EX+NE+EE+SS.BPNS+BPNF	21.339	0.250	0.158	0.181	0.2389	0.479	0.490
1factor	EX+NE+EE+SS+BPNS+BPNF	24.549	0.224	0.130	0.194	0.2357	0.399	0.408
	Criteria(Kline,2018)	< 3	>0.90	>0.90	< 0.05	< 0.05	>0.90	>0.90

EX, extraversion; NE, neuroticism; BPNS, basic psychological need satisfaction; BPNF, basic psychological need frustration; EE, exercise enjoyment; SS, social support.

Table 2 also notes descriptive statistics for reliability and validity tests, and the Pearson correlation matrix of the latent variables in this study. In terms of reliability and Convergent Validity, Cronbach's alpha coefficients for all variables ranged from 0.682 to 0.931. Although the Cronbach's α for neuroticism (0.682) was slightly below the traditional 0.70 threshold, extensive psychometric literature establishes that Cronbach's α is highly sensitive to scale length and severely underestimates the internal consistency of two-item scales (like the BFI-10 subscales). Therefore, relying solely on α in this context is mathematically inappropriate. To provide a robust assessment of reliability, we evaluated the Composite Reliability (CR), which accounts for specific factor loadings. The CR values for all constructs, including neuroticism (CR = 0.913), ranged from 0.728 to 0.932. These robust CR values indicate statistically excellent internal consistency and confirm the reliability of our core independent variables. The Average Variance Extracted (AVE) for all latent variables met the benchmark of values above 0.50 (0.536 to 0.838), which indicates that the scale items provided good variance in the latent measures, and good Convergent Validity (37). In terms of the correlation analysis, the results showed that the directions of relationships among core variables were consistent with theoretical expectations. For extraversion and social support, there were statistically significant positive associations with psychological need satisfaction and exercise enjoyment, while having a statistically significant negative association with psychological need frustration. In opposition, neuroticism displayed a notably dissimilar pattern. Neuroticism showed a statistically significant positive relationship with psychological need frustration, while it exhibited statistically significant negative relationships with psychological need satisfaction and exercise enjoyment. Finally, the positive relationship between psychological need satisfaction and exercise enjoyment, along with the negative relationship between psychological need frustration and exercise enjoyment were established. Together, these findings provide a robust platform for subsequent exploration regarding the inquiry of mediation and moderation processes.

**Table 2:** Description, correlation, reliability, and validity analysis.

Variables	Mean	SD	CR	α	1	2	3	4	5	6
1.Extraversion	5.44	1.64	0.866	0.864	0.764					
2.Neuroticism	2.81	1.79	0.913	0.911	−0.028	0.839				
3.BPNS	5.49	1.42	0.972	0.972	0.304^**^	−0.160^**^	0.746			
4.BPNF	2.86	1.67	0.979	0.979	−0.281^**^	0.514^**^	−0.337^**^	0.797		
5.Exercis enjoyment	5.48	1.57	0.936	0.725	0.348^**^	−0.217^**^	0.337^**^	−0.280^**^	0.785	
6.Social support	5.78	1.27	0.922	0.922	0.241^**^	−0.104^*^	0.138^**^	−0.240^**^	0.217^**^	0.747

The diagonal represents the AVE value. BPNS, basic psychological need satisfaction; BPNF, basic psychological need frustration, ^**^ p < 0.01.

### 3.2 Moderated mediation model for extraversion

**Figure 2:**
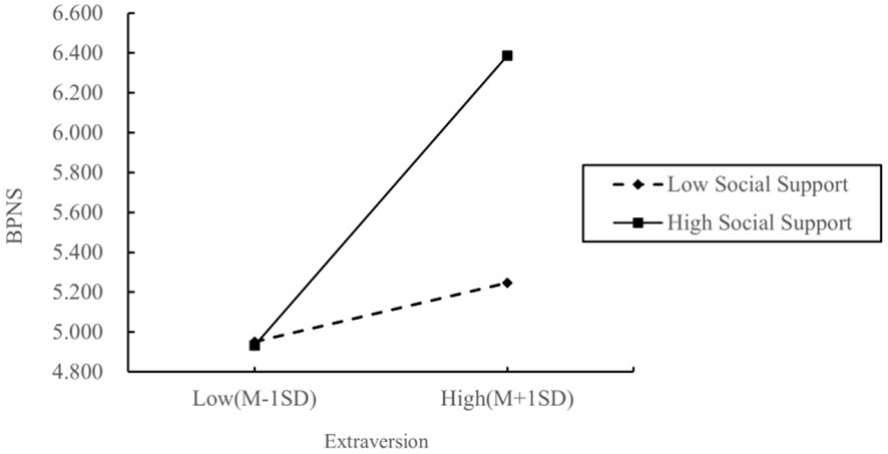
The moderating effect of social support on the relationship between extraversion and BPNS.

As presented in Table 3, regarding the model where psychological need satisfaction served as the outcome variable, extraversion demonstrated a significant positive association with psychological need satisfaction (β = 0.271, *p* < 0.001). Moreover, the interaction term between extraversion and social support was significantly associated with psychological need satisfaction [β = 0.144, *p* < 0.001, 95% CI (0.100, 0.188)]. Simple slope analysis (Figure 2) revealed that when social support was at a low level, the positive link between extraversion and psychological need satisfaction was weaker [β_simple_= 0.090, *p* < 0.05, 95% CI (0.010, 0.171)]. Conversely, when social support was at a high level, this positive link was much stronger [β_simple_= 0.446, *p* < 0.001, 95% CI (0.355, 0.536)], indicating an enhancing pattern. It suggests that high levels of social support are often accompanied by a stronger positive correlation between extraversion and psychological need satisfaction.

**Table 3:** Moderated mediation model (Extraversion).

Predictors	β (Coeff)	SE	t	*p*	LLCI	ULCI
Extraversion → BPNS	0.271	0.034	8.075	0.000	0.205	0.337
Extraversion x Social Support (W) → BPNS	0.144	0.023	6.361	0.000	0.1	0.188
Extraversion → BPNF	−0.258	0.039	−6.576	0.000	−0.335	−0.181
Extraversion x Social Support → BPNF	0.062	0.027	2.331	0.02	0.01	0.114
Extraversion → Exercise enjoyment	0.256	0.037	6.965	0.000	0.184	0.328
BPNS → Exercise enjoyment	0.16	0.043	3.704	0.000	0.075	0.244
BPNF → Exercise enjoyment	−0.155	0.037	−4.215	0.000	−0.228	−0.083
Extraversion x Social Support → Exercise enjoyment	0.131	0.024	5.373	0.000	0.083	0.179
Moderated mediation model						
Extraversion to BPNS at the values of social support (moderator)	Direct effect	SE	*t*	*p*	LLCI	ULCI
M−1SD (-1.252)	0.09	0.041	2.201	0.028	0.01	0.171
M (0)	0.271	0.034	8.075	0.000	0.205	0.337
M+1SD (1.215)	0.446	0.046	9.697	0.000	0.355	0.536
Extraversion to BPNF at the values of social support (moderator)						
M−1SD (-1.252)	−0.335	0.048	−6.973	0.000	−0.43	−0.241
M (0)	−0.258	0.039	−6.576	0.000	−0.335	−0.181
M+1SD (1.215)	−0.183	0.054	−3.401	0.001	−0.289	−0.077
Extraversion (X) to exercise enjoyment at the values of social support (moderator)						
M−1SD (-1.252)	0.092	0.044	2.098	0.036	0.006	0.178
M (0)	0.256	0.037	6.965	0.000	0.184	0.328
M+1SD (1.215)	0.415	0.051	8.182	0.000	0.315	0.514
Mediation models	Boot indirect effect	BootSE	BootLLCI	BootULCI		
Extraversion on exercise enjoyment via BPNS	0.059	0.017	0.029	0.096		
Extraversion on exercise enjoyment via BPNF	0.045	0.015	0.018	0.076		

BPNS, basic psychological need satisfaction; BPNF, basic psychological need frustration, ^***^ p < 0.001.

**Figure 3:**
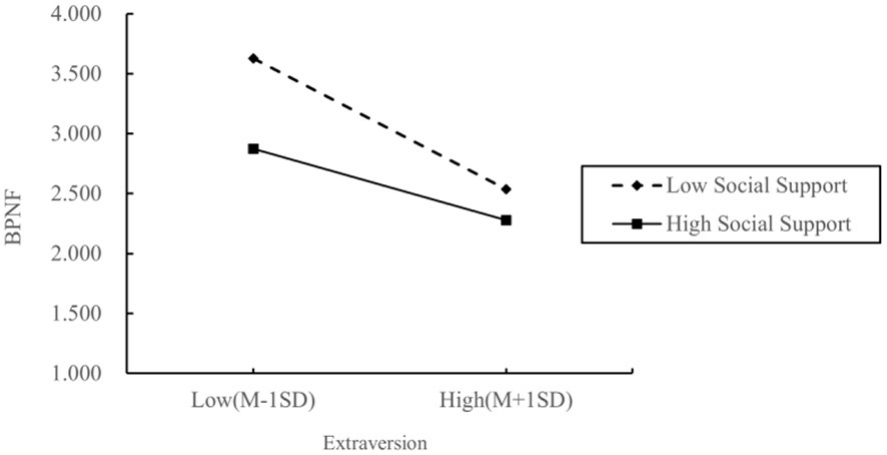
The moderating effect of social support on the relationship between extraversion and BPNF.

Second, in the model with psychological need frustration as the dependent variable, extraversion was negatively correlated with psychological need frustration (β = −0.258, *p* < 0.001). The interaction term also exhibited significance [β = 0.062, *p* < 0.05, 95% CI (0.010, 0.114)]. Simple slope analysis (Figure 3) indicated that at low levels of social support, the negative association between extraversion and lower psychological need frustration was stronger [β_simple_ = −0.335, *p* < 0.001, 95% CI (-0.43,−0.241)]. However, at high levels of social support, although the two variables remained significantly negatively correlated, the strength of the association was attenuated [β_simple_ = −0.183, *p* < 0.01, 95% CI (-0.289,−0.077)]. This implies that in contexts of limited social support, the link between extraversion traits and reduced psychological need frustration is more pronounced.

**Figure 4:**
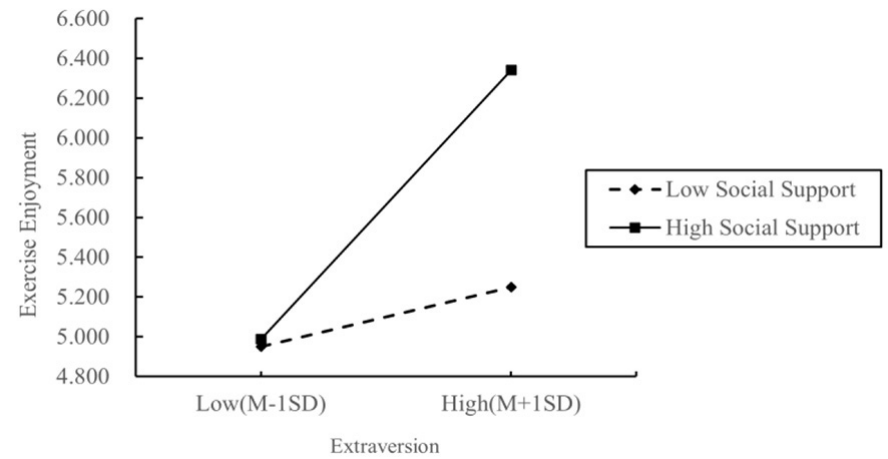
The moderating effect of social support on the relationship between extraversion and exercise enjoyment.

Finally, in the total model accounting for variance in exercise enjoyment, direct associations for all variables were significant. Specifically, extraversion (β = 0.256, *p* < 0.001) and psychological need satisfaction (β = 0.160, *p* < 0.001) were positively correlated with exercise enjoyment, whereas psychological need frustration was negatively correlated with it (β = −0.155, *p* < 0.001). On this basis, the interaction term between extraversion and social support remained significantly associated with exercise enjoyment [β = 0.131, *p* < 0.001, 95% CI (0.083, 0.179)]. Simple slope analysis (Figure 4) again revealed a similar pattern: at low levels of social support, the positive association between extraversion and exercise enjoyment was weaker [β_simple_= 0.092, *p* < 0.05, 95% CI (0.006, 0.178)]. In contrast, at higher levels of social support, the positive relationship between extraversion and exercise enjoyment was significantly amplified [β_simple_ = 0.415, *p* < 0.001, 95% CI (0.315, 0.514)]. This suggests that the positive association between the extraversion trait and exercise enjoyment is most pronounced when individuals have higher social support.

In order to clarify the specific relationships linking extraversion to exercise enjoyment, we utilized the Bootstrap method (with 5,000 re-samples). Specifically, we examined the conditional nature of the indirect relationships via psychological need satisfaction and psychological need frustration as moderated by social support. Initially, without considering the level of the moderator, both mediation models demonstrated significant indirect associations. The indirect links from extraversion to exercise enjoyment were significantly mediated by both psychological need satisfaction [β= 0.059, 95% CI (0.029, 0.096)] and psychological need frustration [β= 0.045, 95% CI (0.018, 0.076)]. This suggests that, in general, the extraversion trait is accompanied by higher psychological need satisfaction and lower psychological need frustration, which in turn are associated with heightened exercise enjoyment.

### 3.3 Moderated mediation model for neuroticism

**Figure 5:**
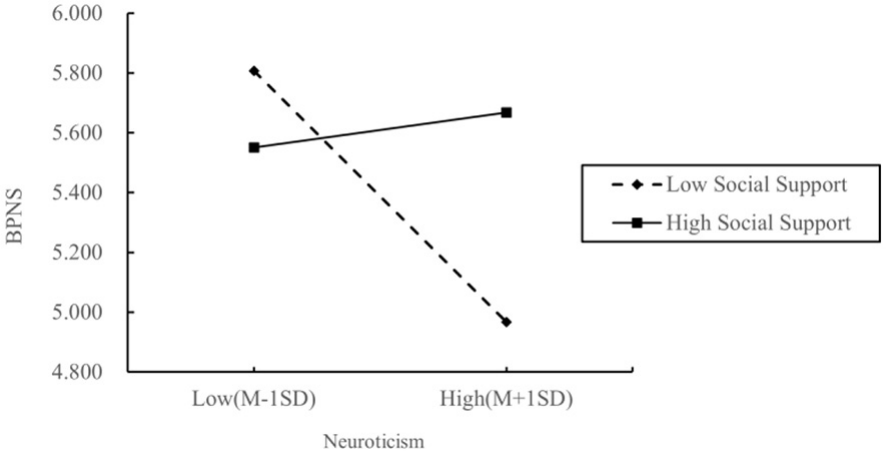
The moderating effect of social support on the relationship between neuroticism and BPNS.

As presented in Table 4 regarding the model where psychological need satisfaction served as the outcome variable, neuroticism demonstrated a significant negative association with psychological need satisfaction (β = −0.098, *p* < 0.01). Simultaneously, the interaction term between neuroticism and social support was significantly associated with psychological need satisfaction [β = 0.107, *p* < 0.001, 95% CI (0.065, 0.150)]. Simple slope analysis (Figure 5) revealed a significant buffering pattern. Specifically, when social support was at a low level, there was a significant negative association between neuroticism and psychological need satisfaction [β_simple_ = −0.233, *p* < 0.001, 95% CI (-0.312,−0.153)]. However, when social support was at a high level, this negative association was no longer significant (β_simple_= 0.032, *p* > 0.05), indicating a buffering dynamic. This suggests that high levels of social support may offset the negative link between neuroticism and psychological need satisfaction.

**Table 4:** Moderated mediation model (Neuroticism).

Predictors	β (Coeff)	SE	*t*	*p*	LLCI	ULCI
Neuroticism → BPNS	−0.098	0.031	−3.18	0.002	−0.159	−0.038
Neuroticism x Social Support → BPNS	0.107	0.022	4.927	0.000	0.065	0.15
Neuroticism → BPNF	0.436	0.031	14.123	0.000	0.376	0.497
Neuroticism x Social Support → BPNF	0.129	0.022	5.926	0.000	0.086	0.172
Neuroticism → Exercise Enjoyment	−0.093	0.036	−2.558	0.011	−0.165	−0.022
BPNS → Exercise Enjoyment	0.246	0.044	5.615	0.000	0.16	0.332
BPNF → Exercise Enjoyment	−0.147	0.044	−3.347	0.001	−0.233	−0.061
Neuroticism x Social Support → Exercise enjoyment	0.069	0.024	2.913	0.004	0.023	0.116
Moderated mediation model						
Neuroticism to BPNS at the values of social support (moderator)	Direct effect	SE	*t*	*p*	LLCI	ULCI
M−1SD (-1.252)	−0.233	0.04	−5.753	0.000	−0.312	−0.153
M (0)	−0.098	0.031	−3.18	0.002	−0.159	−0.038
M+1SD (1.215)	0.032	0.041	0.777	0.437	−0.049	0.114
Neuroticism to BPNF at the values of social support (moderator)						
M−1SD (-1.252)	0.275	0.04	6.795	0.000	0.195	0.354
M (0)	0.436	0.031	14.123	0.000	0.376	0.497
M+1SD (1.215)	0.593	0.041	14.317	0.000	0.512	0.674
Neuroticism to exercise enjoyment at the values of social support (moderator)						
M−1SD (-1.252)	−0.18	0.043	−4.16	0.000	−0.265	−0.095
M (0)	−0.093	0.036	−2.558	0.011	−0.165	−0.022
M+1SD (1.215)	−0.009	0.05	−0.173	0.863	−0.107	0.089
Mediation models	Boot indirect effect	Boot SE	Boot LLCI	Boot ULCI		
Neuroticism on exercise enjoyment via BPNS	−0.033	0.013	−0.061	−0.01		
Neuroticism on exercise enjoyment via BPNF	−0.064	0.022	−0.108	−0.021		

BPNS, basic psychological need satisfaction; BPNF, basic psychological need frustration, ^***^p < 0.001.

**Figure 6:**
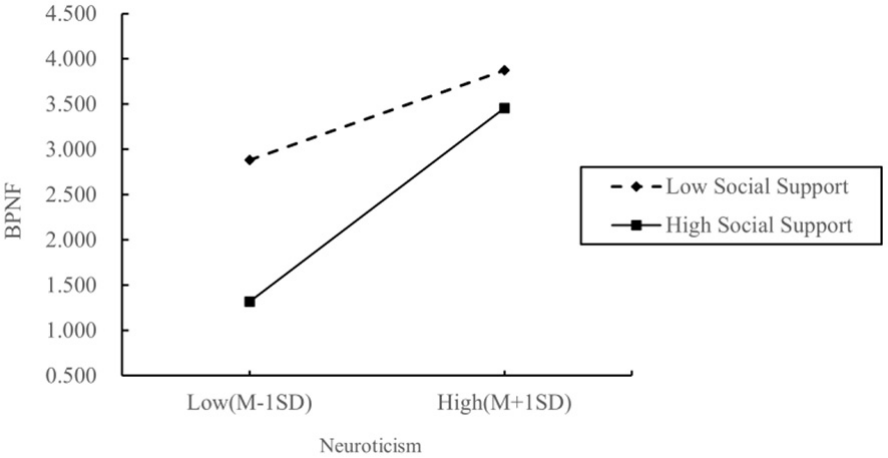
The moderating effect of social support on the relationship between neuroticism and BPNF.

Second, in the model with psychological need frustration as the dependent variable, neuroticism exhibited a very strong positive association with psychological need frustration (β = 0.436, *p* < 0.001). The interaction term also displayed statistical significance [β= 0.129, *p* < 0.001, 95% CI (0.086, 0.172)]. Simple slope analysis (Figure 6) indicated that at low levels of social support, the positive association between neuroticism and psychological need frustration was significant [β_simple_= 0.275, *p* < 0.001, 95% CI (0.195, 0.354)]. Conversely, at high levels of social support, the intensity of this positive association was actually higher [β_simple_= 0.593, *p* < 0.001, 95% CI (0.512, 0.674)].

**Figure 7:**
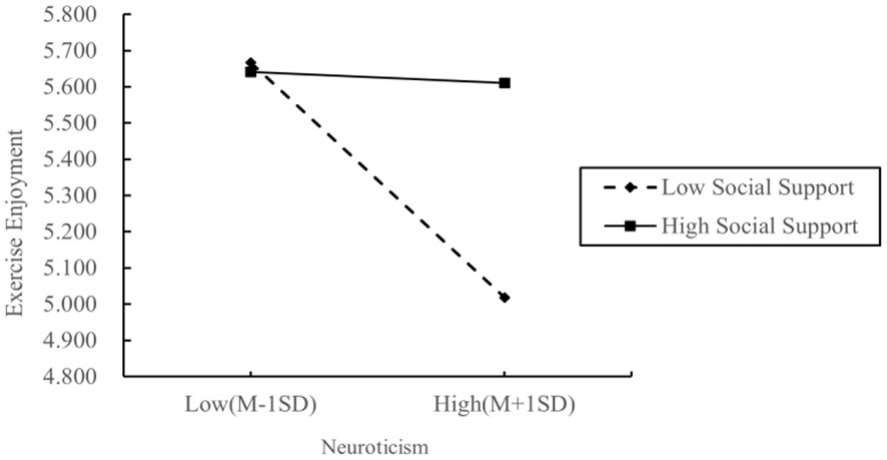
The moderating effect of social support on the relationship between neuroticism and exercise enjoyment.

Finally, in the model explaining variance in exercise enjoyment, direct associations for all variables were significant. Neuroticism (β = −0.093, *p* < 0.05) and psychological need frustration (β = −0.147, *p* < 0.01) were both negatively correlated with exercise enjoyment, whereas psychological need satisfaction was positively correlated with it (β= 0.246, *p* < 0.001). On this basis, the interaction term between neuroticism and social support remained significantly associated with exercise enjoyment [β = 0.069, *p* < 0.05, 95% CI (0.023, 0.116)]. Simple slope analysis (Figure 7) further confirmed the critical protective role of social support. At low levels of social support, neuroticism showed a significant negative correlation with exercise enjoyment [β_simple_ = −0.18, *p* < 0.001, 95% CI (-0.265,−0.095)]. In other words, higher levels of neuroticism were linked to lower exercise enjoyment. However, at high levels of social support, the negative association between neuroticism and exercise enjoyment was not significant (β_simple_ = −0.009, *p* > 0.05). This implies that ample social support appears capable of fully blocking the adverse link between neuroticism and exercise enjoyment.

Similarly, the Bootstrap method (with 5,000 resamples) was utilized to examine the mediation models via psychological need satisfaction and psychological need frustration. This analysis further evaluated the moderated mediation relationships governed by social support. Both mediation models exhibited significant negative indirect associations. The indirect associations between neuroticism and exercise enjoyment were significantly mediated by psychological need satisfaction [β = −0.033, 95% CI (-0.061,−0.010)] and psychological need frustration [β = −0.064, 95% CI (-0.108,−0.021)]. This suggests that, generally, high neuroticism traits are accompanied by lower psychological need satisfaction and higher psychological need frustration. These conditions, in turn, are associated with reduced exercise enjoyment.

## 4 Discussion

Based on the SDT, the present study aimed to develop a moderated mediation model to clarify how extraversion and neuroticism are associated with outdoor exercise enjoyment through both mechanisms of satisfaction and frustration of psychological needs, and to explore the moderation role of social support on these relationships. The findings provide support for the dual-process model. Findings indicated that extraversion and neuroticism were associated with exercise enjoyment through the facilitator link of psychological need satisfaction and the impediment link of psychological need frustration. Significantly, our findings revealed that social support can serve in distinctly different ways dependent on individual differences in personality, a booster for extraversion and a buffer for those high in neuroticism. Social support may also emerge as a potential stressor in some of the specific pathways.

### 4.1 Validation of the dual mediation mechanisms associated with exercise enjoyment

Findings from this study are consistent with earlier findings around relationships between personality and physical activity (38, 39), with potentially more illuminating findings emerging through the asymmetric roles of psychological need satisfaction and psychological need frustration. Data tended to support the notion that psychological need satisfaction and psychological need frustration are not oppositional elements on a continuum, but rather two distinct processes (10). Extraversion appeared to be associated with high enjoyment via the bright side link of enhancing psychological need satisfaction, possibly in a manner that reflects the reward sensitivity of extraversion (16). In contrast, the strong association between neuroticism and psychological need frustration (18) suggests a different mechanism. For individuals high in neuroticism, enjoyment deficits may be associated with dark side mechanisms related to feelings of incompetence or rejection, rather than the absence of satisfaction (12).

The study aims to further the conversation around psychological need frustration and negative outcomes (9), into the discourse on personality differences. Our findings suggest that low exercise enjoyment among highly neurotic individuals may be related to tendencies toward negative self-talk or threat appraisal in stressful situations (40). This also somewhat supports the more general notion that positive and negative affect may be generated by separate drives and further reinforces calls by academics to consider more closely affective experiences (41, 42). we are limited to descriptive analysis and cannot draw causal directions from the findings as this was a cross-sectional study. As temperament and behaviors may interact in a more complex bidirectional manner (13), for instance, it is equally plausible that chronically low exercise enjoyment could reciprocally exacerbate neurotic tendencies over time, caution must be exercised in attributing directional relationships among these variables.

### 4.2 The moderation paradox of social support: from beneficial gains to a double-edged sword

This study highlights a more sophisticated portrait concerning the moderating role of social support. Social support appeared to enhance the positive relationship between extraversion and psychological need satisfaction, likely functioning as an important facilitatory resource, a gain association (22). The finding may relate to the greater socially oriented extract resources present in extraversion (43). These themes likely reflect a positive person-environment fit (44). For neuroticism, results present some duality. Social support was associated with a weaker negative relationship between neuroticism and psychological need satisfaction. This finding is somewhat supportive of the stress-buffering hypothesis (45, 46); social support may function as a psychological shielding mechanism for vulnerable individuals.

Alternatively, a paradox that requires careful consideration emerged: in conditions of elevated social support, the positive relationship between neuroticism and psychological need frustration appeared to increase. This counter-intuitive observation suggests several potential mechanisms. First, as our social support measure assesses perceived quantity rather than quality (e.g., autonomy-supportive vs. controlling), we can only speculate that within the specific socio-cultural context of China's first-tier cities—characterized by a hyper-competitive social climate, often described as involution (neijuan)—social support from close ties might take on controlling characteristics. In this specific milieu, excessive support may be appraised by hypersensitive neurotic individuals as a form of evaluative surveillance or an implicit expectation to succeed (7, 47–49). Alternatively, and equally importantly, this paradox may not stem from the nature of the support itself, but rather from the inherent cognitive tendencies of neuroticism. Individuals high in neuroticism often exhibit a negative interpretive bias, potentially misconstruing well-intentioned support as intrusive or patronizing. Furthermore, the observed association might reflect a distress-driven process, wherein individuals experiencing high need frustration actively seek out more support as a coping mechanism, rather than the support causing the frustration.

### 4.3 Person-environment fit within a competitive urban context and differentiated interventions

The divergent associations of social support across different personality traits provide a nuanced perspective on Person-Environment Fit theory. Originating from China's first-tier cities, the sample for this study is situated within a highly dense and competitive urban environment. Rather than attributing our findings to broad cultural differences, this specific socio-environmental context provides a critical grounded lens for understanding interpersonal interactions. In relation to social support, the benefit for extraversion may demonstrate how social networks formed via close social support effectively fulfill psychological needs of relatedness (49). For neurotic individuals, however, heightened frustration is likely linked to intense social evaluation anxiety, social comparison pressure, and peer conformity pressures prevalent in highly competitive urban settings, rather than abstract cultural norms. Subjective experience often takes precedence over objective form, and in this case, support with respect to rigid group expectations may be viewed as a decrease in autonomy (50, 51). Additionally, in the Chinese context, support from strong ties, if done poorly, may result in more interpersonal stress than weak ties (52). Thus, while acknowledging the relatively modest effect sizes observed in our moderation models, differentiated micro-level strategies may still offer practical value. For extraversion, communities should be encouraged to maximize psychological need satisfaction (53). For neuroticism, the intervention focus should shift from the amount of social interaction to the quality of interaction. Engendering a non-judgmental, mindfulness-based environment may be more important. This approach aims to lessen social evaluation anxiety, which may interrupt the pathway to psychological need frustration (18, 54). Additionally, implementing low-competition designs based on habit formation perspectives may reduce barriers to participation (55). While it is acknowledged that the cross-sectional design of this study hampers capturing ongoing changes in environmental adaptation, it is suggested that future research should consider how macro-environmental pressures moderate personality and exercise experiences over time (46).

## 5 Conclusion

This study investigated the complex psychological mechanisms driving outdoor exercise enjoyment by examining the dual, asymmetrical associations of basic psychological needs and the moderating role of social support. We conclude that personality traits significantly dictate exercise experiences: extraversion facilitates enjoyment primarily through the satisfaction of psychological needs, whereas neuroticism diminishes enjoyment largely through the frustration of these needs. Crucially, this study challenges the traditional “universally beneficial” assumption of social support. We conclude that social support is highly conditional. While it acts as a valuable resource that amplifies need satisfaction for extraverted individuals, it can paradoxically function as a stressor that exacerbates need frustration for highly neurotic individuals, particularly within competitive social contexts. Ultimately, this research underscores the necessity of moving beyond a “one-size-fits-all” approach, advocating for tailored, personality-informed strategies to promote sustained outdoor physical activity.

## 6 Limitations and future directions

This study has several limitations that inform future research directions. First, by focusing exclusively on extraversion and neuroticism via the abbreviated BFI-10 scale, this study conceptually aligns with our SDT framework but inherently limits the multidimensional assessment of personality. Although the reliability of our abbreviated measurement was statistically validated by both a strong Spearman-Brown coefficient (ρsb = 0.911) and Composite Reliability (CR = 0.913) despite the naturally marginal Cronbach's α, future studies should employ comprehensive inventories (e.g., NEO-FFI) to explore the unique roles of other traits (e.g., conscientiousness) in the dual-need relationships. Second, the cross-sectional design precludes causal inferences, and our measures primarily focused on the sources rather than the qualitative functions of social support (e.g., autonomy-supportive vs. controlling). Future research should utilize longitudinal or ecological momentary assessment (EMA) designs, supplemented by qualitative interviews, to unravel the bidirectional causal chains and pinpoint why support may trigger frustration. Third, it is important to acknowledge that the explained variance in our moderation models is relatively modest. While the interaction terms are statistically significant, these small overall effect sizes indicate that outdoor exercise enjoyment is a multifaceted phenomenon influenced by numerous unmeasured environmental and intrapersonal variables. Consequently, our findings should be interpreted cautiously as offering nuanced guidance rather than sweeping policy interventions. Finally, our sample predominantly comprised highly educated individuals in China's first-tier cities, presenting an “elite bias.” As discussed, this highly competitive urban environment is likely the specific catalyst triggering the “stress response” to social support among neurotic individuals. To ascertain whether this moderation paradox generalizes to less evaluative or rural settings, future research must implement cross-regional comparative designs to validate the boundary conditions of these psychological associations.

## Data Availability

The original contributions presented in the study are included in the article/Supplementary material, further inquiries can be directed to the corresponding author.
