# Supplementary figures and images for: Beyond a one-size-fits-all approach: the asymmetrical roles of personality and social support in promoting outdoor physical activity
Source: Front Public Health. 2026 Jul 15;14:1820567. doi: 10.3389/fpubh.2026.1820567 (PMC13414837; doi:10.3389/fpubh.2026.1820567)

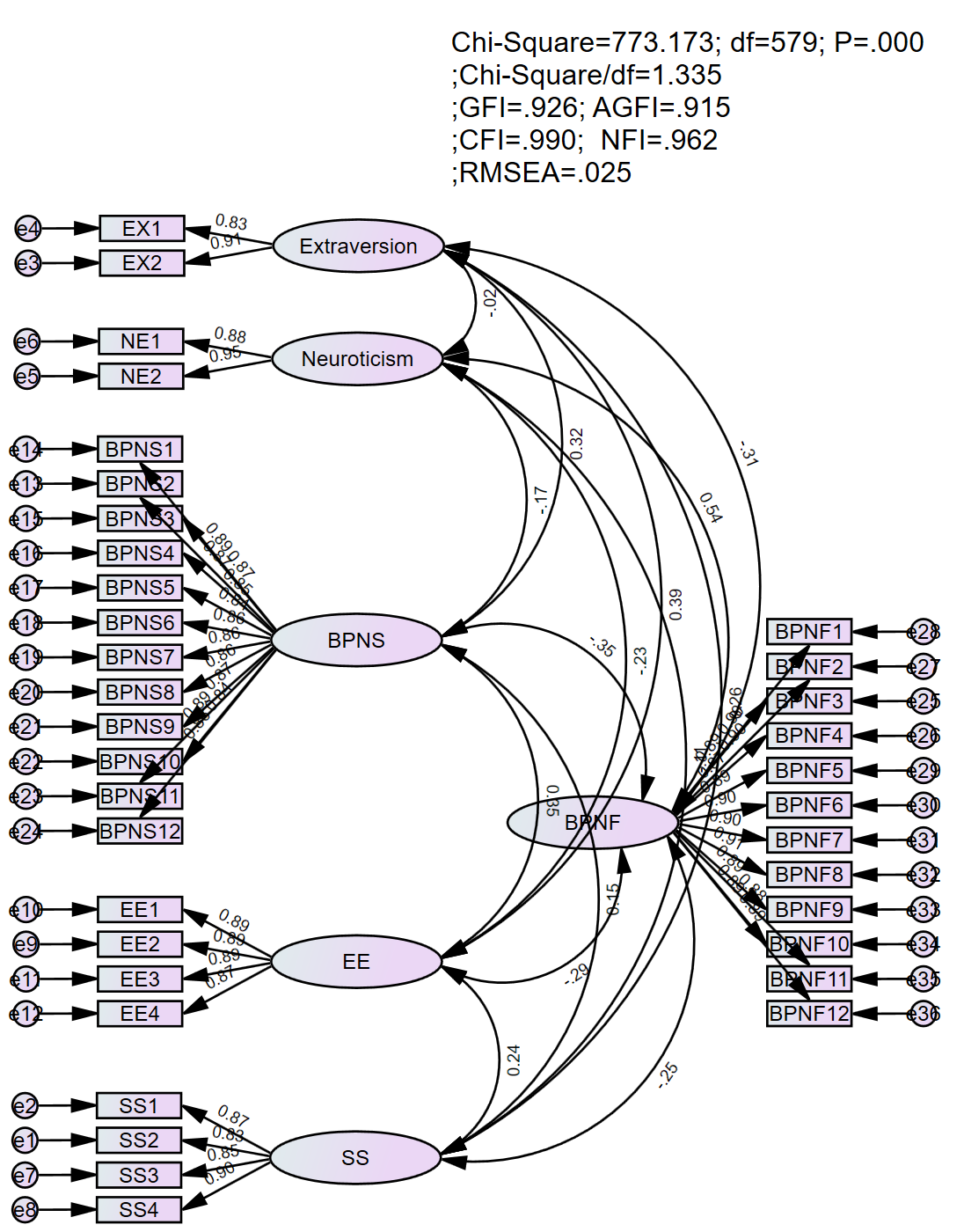
Appendix

Supplement: Supplementary file 1 [file Supplementary_file_1.docx]
